# The Promise of Personalized Peripheral Nerve Surgery

**DOI:** 10.3390/jpm14060608

**Published:** 2024-06-07

**Authors:** Konstantin Davide Bergmeister, Leila Harhaus

**Affiliations:** 1 Clinical Laboratory for Bionic Extremity Reconstruction, Department of Plastic, Reconstructive and Aesthetic Surgery, Medical University of Vienna, Vienna, Austria; 2 Department of Plastic, Aesthetic and Reconstructive Surgery, Karl Landsteiner University of Health Sciences, University Hospital St. Poelten, 3100 Krems, Austria; 3 Department of Hand-, Plastic and Reconstructive Surgery, Burn Center, BG Trauma Center Ludwigshafen, Department of Hand- and Plastic Surgery, University of Heidelberg, Heidelberg, Germany; leila.harhaus@bgu-ludwigshafen.de; 4 Department of Hand Surgery, Peripheral Nerve Surgery and Rehabilitation, BG Trauma Center, Ludwigshafen, Germany

In peripheral nerve surgery, neuropathology and neural anatomy intersect with the complexities of injury and dysfunction [1,2]. Here, the quest for personalized care has emerged as a guiding principle for better outcomes [3]. As clinicians and researchers alike strive to optimize patient outcomes, a diverse array of innovations have taken center stage, from modern diagnostic modalities to novel surgical innovations. At the heart of this transformative journey lies the idea of treating patients in a personalized manner to best suit their pathology and individual needs [4].

The landscape of personalized peripheral nerve surgery is illuminated by a number of research endeavors, each shedding light on different facets of nerve diagnostics and therapeutic interventions. This Special Issue on the topic of peripheral nerve surgery published in the *Journal of Personalized Medicine* serves as a testament to this collective effort, featuring a rich variety of studies exploring both diagnostic and therapeutic strategies aimed at optimizing patient care.

At the forefront of this transformative landscape are novel diagnostic techniques such as MR-neurography and nerve ultrasound, which offer unprecedented insights into nerve anatomy, pathology, and function [5,6]. MR-neurography, a specialized MRI technique tailored to visualize peripheral nerves with high spatial resolution, enables clinicians to identify nerve injuries, compressions, and anatomical variants with remarkable clarity [7]. By employing advanced imaging sequences and protocols optimized for nerve tissue, radiologists can delineate the course and integrity of peripheral nerves, facilitating targeted interventions and personalized treatment plans.

Similarly, nerve ultrasound provides real-time visualization of peripheral nerves at the point of care, offering valuable insights into nerve morphology, vascularity, and dynamic function [8]. From detecting common entrapment syndromes to identifying nerve lesions, nerve ultrasound serves as a versatile tool for diagnosing and managing a wide spectrum of peripheral nerve disorders. In contrast to MR-neurography, nerve ultrasound has become more and more available, even for treating clinicians, allowing easy point-of-care use whenever needed.

In addition to advancements in diagnostic imaging, innovative or newly explored surgical techniques are reshaping the landscape of peripheral nerve surgery, offering new avenues for nerve reconstruction and functional restoration [9]. For example, one such idea is the use of muscle-in-vein conduits for the treatment of symptomatic neuroma of sensory digital nerves [10]. Alternatively, costovertebral exarticulation of the first rib may be a viable alternative in patients with neurogenic thoracic outlet syndrome, as demonstrated in a retrospective clinical study [11]. Furthermore, research on nerve transfers for brachial plexus reconstruction in patients over 60 years underscores the importance of age-specific considerations in surgical decision-making [12]. By tailoring treatment strategies to the unique needs and physiological characteristics of older patients, clinicians can optimize outcomes and enhance quality of life, reaffirming the principle of personalized care in peripheral nerve surgery.

In conclusion, the journey towards personalized peripheral nerve surgery is characterized by innovation [13,14], collaboration [15], and a relentless pursuit of excellence. As we harness the power of novel diagnostic modalities and surgical innovations [16], we move closer to our ultimate goal, i.e., delivering precision care that is tailored to the unique needs of each patient. By better understanding nerve pathology and leveraging cutting-edge technologies, we can personalize treatment approaches, optimize outcomes, and improve the lives of individuals affected by nerve injuries and compression syndromes. In essence, a better diagnosis of nerve lesions leads to better treatment, underscoring the transformative potential of personalized peripheral nerve surgery in shaping the future of healthcare.
